# Topic evolution and sentiment comparison of user reviews on an online medical platform in response to COVID-19: taking review data of Haodf.com as an example

**DOI:** 10.3389/fpubh.2023.1088119

**Published:** 2023-06-02

**Authors:** Chaoyang Li, Shengyu Li, Jianfeng Yang, Jingmei Wang, Yiqing Lv

**Affiliations:** ^1^School of Management, Henan University of Technology, Zhengzhou, China; ^2^Business School, Zhengzhou University, Zhengzhou, China

**Keywords:** online medical platform, Haodf website, text mining, topic analysis, sentiment analysis, COVID-19 pandemics

## Abstract

**Introduction:**

Throughout the COVID-19 pandemic, many patients have sought medical advice on online medical platforms. Review data have become an essential reference point for supporting users in selecting doctors. As the research object, this study considered Haodf.com, a well-known e-consultation website in China.

**Methods:**

This study examines the topics and sentimental change rules of user review texts from a temporal perspective. We also compared the topics and sentimental change characteristics of user review texts before and after the COVID-19 pandemic. First, 323,519 review data points about 2,122 doctors on Haodf.com were crawled using Python from 2017 to 2022. Subsequently, we employed the latent Dirichlet allocation method to cluster topics and the ROST content mining software to analyze user sentiments. Second, according to the results of the perplexity calculation, we divided text data into five topics: diagnosis and treatment attitude, medical skills and ethics, treatment effect, treatment scheme, and treatment process. Finally, we identified the most important topics and their trends over time.

**Results:**

Users primarily focused on diagnosis and treatment attitude, with medical skills and ethics being the second-most important topic among users. As time progressed, the attention paid by users to diagnosis and treatment attitude increased—especially during the COVID-19 outbreak in 2020, when attention to diagnosis and treatment attitude increased significantly. User attention to the topic of medical skills and ethics began to decline during the COVID-19 outbreak, while attention to treatment effect and scheme generally showed a downward trend from 2017 to 2022. User attention to the treatment process exhibited a declining tendency before the COVID-19 outbreak, but increased after. Regarding sentiment analysis, most users exhibited a high degree of satisfaction for online medical services. However, positive user sentiments showed a downward trend over time, especially after the COVID-19 outbreak.

**Discussion:**

This study has reference value for assisting user choice regarding medical treatment, decision-making by doctors, and online medical platform design.

## 1. Introduction

Online medical platforms utilize information technology such as the Internet to expand the availability and breadth of medical services (1). Especially in under-resourced areas or in the context of major public health emergencies, online medical services have become an indispensable medical supply channel. Many online medical platforms have sprung up in China, such as Chunyuyisheng.com and Haodf.com, both of which help users obtain medical services in some capacity (2). According to the 50th Statistical Report released by the China Internet Network Information Center, the number of online medical users reached 300 million as of June 2022, marking an increase of 1.96 million from the previous year and accounting for 28.5% of Internet users.

Information asymmetry between doctors and patients persists in online medical services (3). Online medical reviews contain feedback comments from users (4), which record users’ evaluations of doctors, medical skills, treatment effects, and other services, as well as users’ experiences, rehabilitation processes, feelings, and mental states. Such rich user information can reduce information asymmetry (5). Consequently, when selecting a doctor, users often refer to reviews on online platforms. Therefore, the acquisition and processing of such information can allow researchers to evaluate the level of medical services comprehensively and accurately (6).

Although online reviews provide a reference for users to support decision-making, the content of reviews differs over time because the status and service quality of online medical platforms varies temporally. Currently, the online medical service market in China has made great achievements. In fact, the industry has gone through three stages: early start, standardized development, and accelerated progress after the COVID-19 pandemic. In the initial phase, the Haodf.com platform was founded in 2006. Subsequently, an increasing number of platforms emerged, such as Chunyu Doctor, Ping An Good Doctor, Ali Health, and Jingdong Health. However, this stage was characterized by a lack of supervision.

The national medical and health regulatory authorities later strengthened supervising efforts in the emerging online healthcare market. From 2017 to 2020, the development of the online medicine industry slowed down significantly and entered the standard development stage. In 2018, several important policies and regulations were issued, which were critical for market development. In September, the National Health Commission officially issued the Administrative Measures for Internet Diagnosis and Treatment (on a trial basis), which provided basic normative guidance for the online healthcare industry. A couple of years later, 2020 was a turning point for online healthcare industry. Due to the societal and healthcare impacts of COVID-19, the government issued a series of online healthcare industry norms to improve business environments and facilitate high-quality development. These policies accelerated the popularization of online medical services.

The government is constantly implementing and revising regulatory methods for the online medical platform market; service quality also tends to improve gradually. However, due to the impact of the pandemic and other factors, users’ habits and demands for online medical services are constantly changing (7). In other words, with the normative development of medical platforms and change in user demand being at different stages, the content and sentiments captured in user reviews on online medical services will vary over time. Examining changes to online review data over time elucidates a new perspective for analyzing user demand and changes to online medical platform quality.

By mining the topics and sentiments of online reviews at different stages, we may understand the changing rules of user demand for online medical services and evaluate changes in online medical platform service quality in China (3). Although China is the world’s largest online medical service market, very few papers have explored online medical reviews over time. Considering this, we analyzed review data extracted from a major online medical platform from a time series perspective. This study explored the topics and sentimental characteristics of user reviews in different time stages by extracting user-generated content. We also focused on the changing characteristics of medical service topics and sentiment before and after the COVID-19 pandemic, which we believed would elucidate the changing rules of user needs under the impact of the COVID-19 pandemic. This focus is expected to provide a data reference for governments, medical platforms, and other parties.

The remainder of this paper is arranged as follows: the second section is the literature review; the third section introduces materials and methods; the fourth section details the results of our analysis; and the fifth section discusses the findings, details our conclusions, and sets forth policy recommendations.

## 2. Literature review

### 2.1. Online medical platforms

Doctors play a central role in the online medical community, and numerous studies have been discussed from the point of view of doctors. Most of the research in this area focuses on the extrinsic, intrinsic, and professional motivations of physicians’ performance on an online healthcare service platform (8). Word of mouth can incentivize physicians to improve efficiency and service on online healthcare service platforms. Doctors can be incentivized by word of mouth to improve efficiency and service on online healthcare service platforms. Zhang et al. (9) studied the relationship between doctors’ personal online brand strategies, environment and performance. A doctor’s brand image has the potential to significantly improve brand performance. Yang et al. (10) focused on the relationship between the quality of online physician services and patient satisfaction. They find that a doctor’s speed of response has a very positive influence on patient satisfaction. The frequency of interaction between a doctor and a patient also has a positive effect on patient s satisfaction. Wu et al. (11) found that the quality of doctor service significantly affected user praise and that this effect varied over time. Shah et al. (12) investigated the relationship between online informational signals and physicians’ economic performance. Their research shows that signals of the quality of doctors’ services and the trustworthiness of doctors significantly improve patients’ willingness to pay a price premium. Wan et al. (3) factors affecting doctors’ consultation volumes. Their research found that physicians’ soft skills such as reputation, phrasing, and trustworthiness affect the volumes of physician visits and patient satisfaction.

Online medical teams (OMT) are wildly popular as a new form of online medical consultation platform. Some academics explored the link between patient satisfaction and doctors’ characteristics from a team perspective. Yang et al. (13) studied how team structure, such as diversity of titles, diversity of reputation, and diversity of services, affect team engagement. Wang et al. (14) further strengthened the evidence linking individual-and team-level professional capital to physicians’ performance through a cross-level model. They found that the status capital of doctors and the decisional capital of an OMT can increase doctors’ performance. Their study provides insight into physicians’ performance from an intersectional perspective. Liu et al. (15) found that current research in this area needs to be more clearly explicated. Extending the theory of self-determination, their research analyzed how physician-and team-specific motivators positively impact physician engagement. Social interaction has been found to have a positive relationship with physician engagement, whereas team size will weaken this positive promotion as team size is negatively related to social interaction ties.

Disease is a changing and dynamic medical problem, and patients’ feelings are different at different points in time, even for the same illness. The patient’s need for a physician will change depending on different scenarios (16). Patients want to avoid risk by selecting doctors with good reputations (17). When selecting physicians, patients will seek physicians’ online information (9). For example, Liu et al. (17) found that patients often rated physicians’ service performance based on the physicians’ online recommendation when choosing a physician for further consultation. Through this service, patients and physicians can build sustainable online relationships on the platform. Online relationships are different from traditional patient-physician relationships. However, the disease treatment plan still depends on professional advice from doctors (16). Patients must still face the disadvantage of asymmetric information. Zhang et al. (18) investigated interactional injustice online in the quality of patient–doctor relationships. They found that interpersonal injustice online negatively affects relationship quality incentives.

Once the physician has completed the online service, patients can provide feedback to the consulting physician. Hao (19) analyzed reviews on Haodf.com and concluded that user reviews were primarily positive, which reflected findings reported regarding user reviews in the United States and Europe. The study also found that Chinese users were increasingly utilizing comments as an important reference when selecting doctors. Meng et al. (20) considered the impact of physician reputations and user behaviors on platform knowledge sharing. Ma et al. (21) discussed the information needs of users with cancer and their families on online medical platforms as well as the potential influence of Chinese culture on information needs. Chen et al. (6)studied the influence of online doctor–patient interactions on user satisfaction, and the results showed that user activity positively impacted doctors’ information and emotional support.

An alternative source of health information for patients is the online health consultation, which can assist patients in obtaining professional suggestions from online physicians (22). Online medical consultations can better address the shortage of medical and healthcare services (23, 24). There has been a continuous increase in research in this area. Breaking time and location limits allows patients to schedule appointments and see their doctors online. Through online medical consultation, older adults with disabilities or who live in rural areas are better able to meet the demand for medical services, which can improve public health (16).

A number of studies have focused on the benefits and mechanisms of online health communities such as platform characteristics, levels of customer satisfaction and regulations (6). With the emergence of healthcare service platforms, the problem of information asymmetry can be effectively alleviated and effective information can be provided to patients (3). Online platforms can promote patients’ trust and confidence in physicians. In the online healthcare service environment, doctors are stimulated to improve their brand images (9). Patients have a wide range of choices in selecting doctors. Patients have a wide range of physician selection choices. In order to develop an online healthcare service platform, many doctors need to be attracted who can actively provide medical services (15).

Liu (25) reported the advantages and disadvantages of online medical platforms through topic and sentimental analysis of reviews on Haodf.com. Zhang et al. (26) collected negative comments on Haodf.com and made suggestions to improve both user satisfaction and the relationship between users and doctors. Hao et al. (27) compared the health systems of the United States and China by using reviews of obstetrics and gynecology on RateMDs.com and Haodf.com, respectively. Since there are two sides to a coin, online medical platforms have both their advantages and disadvantages. Several ethical and legal issues related to the practice of online medical services remain unresolved and in need of proper regulation (28).

### 2.2. Text mining

Text mining is the most basic method for extracting meaningful information from review texts (29) and is an increasingly common research technique (30). For example, Ludwig et al. (31) analyzed customer conversion rate from sentimental content and language style changes of online comments by mining review text. Zhang (32) used ROST software to mine comments on relevant tourism industries and concluded that the management department of scenic areas should focus on the integration of “tourism+” industry. Wu and Yao (33) collected text from online reviews by tourists and conducted sentiment analysis with ROST software to evaluate their satisfaction with tourist attractions. Qi and Liu (34) mined the review text on the MOOC platform and investigated learner attitudes regarding online courses. Their findings provide some references for students and educators.

The topic model is a prominent machine learning technology that is widely used in text mining and data value discovery (29). Due to the high scalability of latent Dirichlet allocation (LDA) in topic modeling (35, 36), it has become one of the most popular methods in this field. For example, Xue et al. (37) used the LDA model to analyze public sentiments toward 11 topics related to the COVID-19 pandemic in tweets. Tirunillai et al. (38) mined product review data to explore product competition through LDA modeling. Yan and Shao (39) studied how opinion leaders effectively utilize bullet screens to carry out marketing activities on social media platforms. Their research was based on mining review information of the video sharing website Billili.com and building an LDA model through text feature extraction and text clustering. Gorro et al. (40) collected responses to COVID-19 from Filipino citizens on Facebook using LDA modeling. After the outbreak of COVID-19, the number of studies using LDA increased dramatically (41).

## 3. Materials and methods

**Figure 1:**
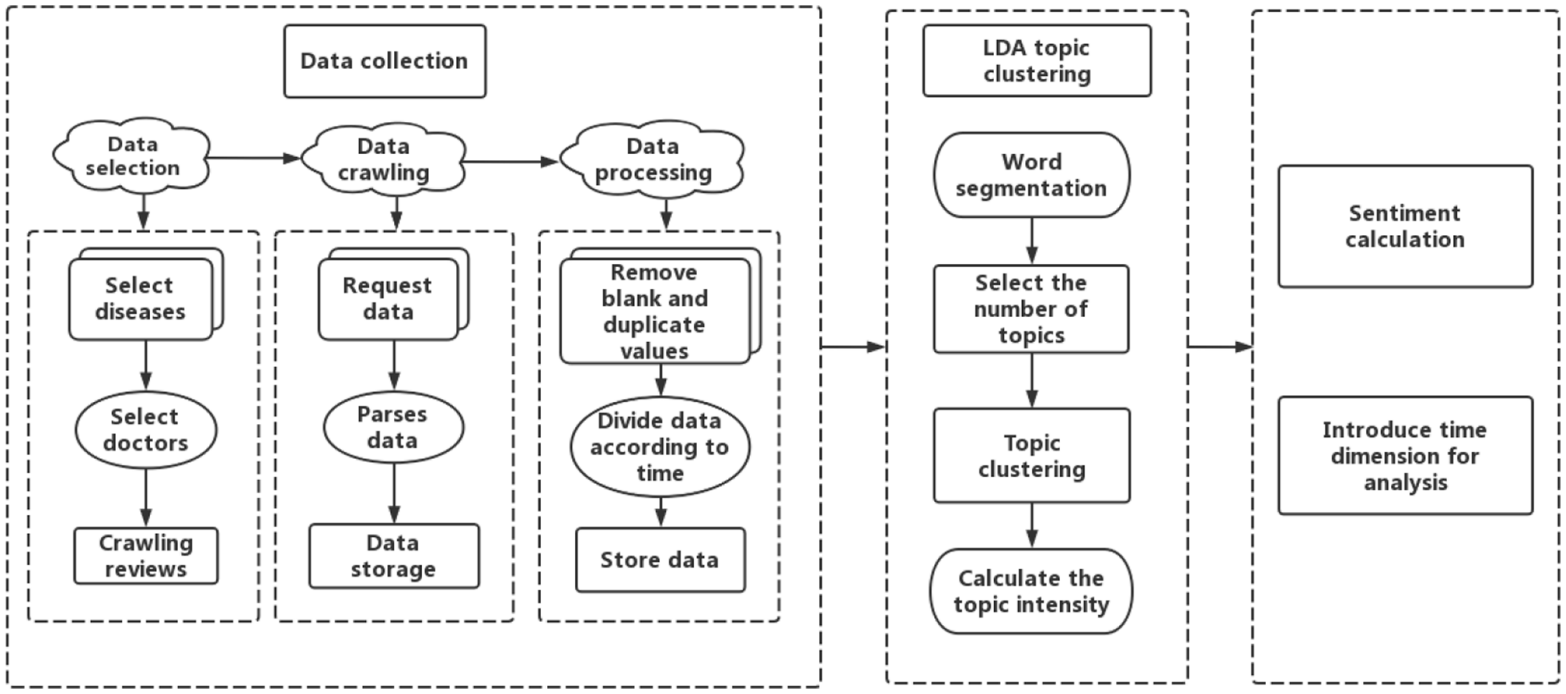
Research framework.

Our research process is shown in Figure 1. In this study, user review data from the online medical platform Haodf.com was mined using an LDA model and ROST content mining (CM) software. Subsequently, we analyzed changes in topics and sentiments in reviews on the platform over time. The research process followed four primary steps:

1. Python software was used to collect user review texts in chronological order. These data were preprocessed by removing duplicate and null values.
2. Feature clustering of review texts was obtained using an LDA topic model, and topics were extracted.
3. ROST CM software was used to analyze the sentiments captured in review texts.
4. Topics and sentiments in review texts were analyzed across different time periods.

### 3.1. Data collection and preprocessing

#### 3.1.1. Platform selection

Haodf.com, a medical consulting platform in China, was selected as the target online medical platform in this study. The Haodf.com platform was founded in 2006 and is one of the leading online medical platforms in China. As of July 2022, 890,000 doctors from more than 10,000 hospitals in China were registered on the platform. Among them, 250,000 doctors were registered by name and could directly provide online medical services to users. The percentage of these active doctors in first class hospitals was 73%. Hence, this platform is considered to have high medical service authority and was considered suitably representative of user engagement with online medical platforms.

#### 3.1.2. Review text acquisition process

The text of user reviews was obtained using Python. During the data crawling step, we first used the Requests package in Python to crawl HTML data from doctors’ homepages. Then, using the requests package in Python, we parsed the crawled HTML data. Finally, we saved the parsed data. To ensure the robustness of our research, in our study, 44 common diseases were selected from 4 broad categories. In the first category are acute illnesses, such as poisoning, cerebral infarction, atrial fibrillation, and so on. Then there are chronic diseases such as diabetes, rheumatism, coronary heart disease and so on. Mild illnesses, such as colds, dizziness, coughs, etc., fall into the third category. The second is chronic diseases like diabetes, rheumatism, coronary heart disease, etc. The fourth category is severe, such as cancer, tumor, leukemia, etc. We select physicians with high volumes of visits from well-known hospitals in China, such as Xiangya Hospital Central South University, Peking University First Hospital, Peking Union Medical College Hospital, and so on. Finally, we collected data from 2,122 physicians.

Here is the detailed collection of comment text: first, we used Python to crawl the links related to the selected 44 diseases into the CSV file. Second, according to pages linked to the target diseases, the software crawled data from the pages of linked doctors. Because a doctor may treat multiple disease types, the doctor’s homepage may have been associated with different links across multiple diseases. Therefore, we needed to remove duplicate links for each doctor. Finally, comments for 2,122 doctors were obtained. A total of 433,288 review texts were crawled for this study. To ensure the objectivity of the collected data, we preprocessed the crawled data by removing duplicate values and subsequently obtained 323,519 review texts from 2017 to 2022.

### 3.2. Topic extraction and intensity calculation

#### 3.2.1. Topic extraction based on LDA model

**Figure 2:**
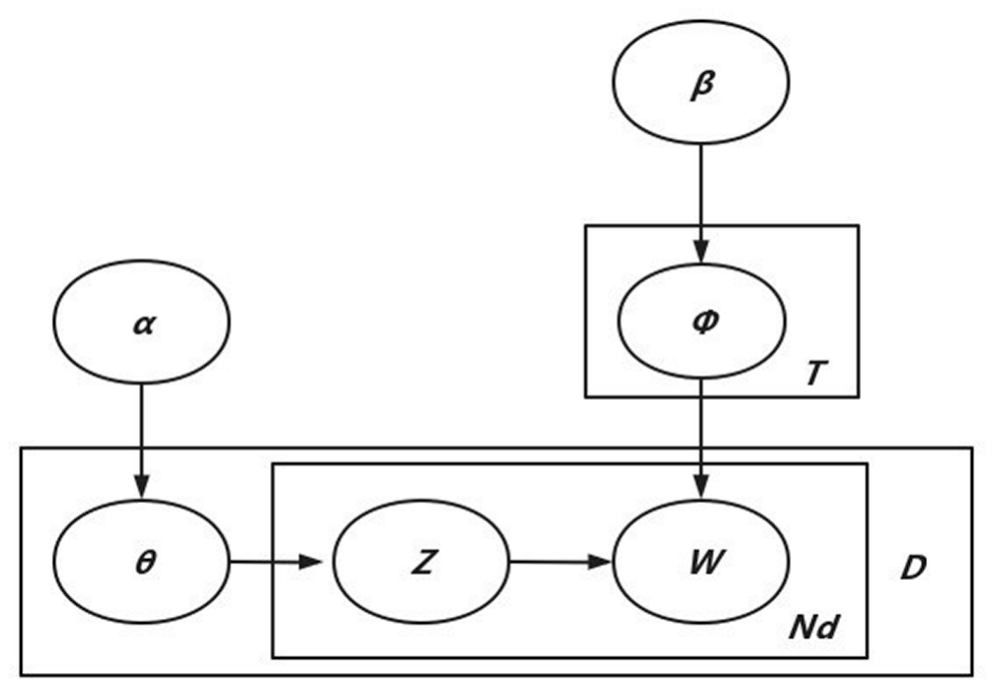
LDA model flow chart.

An LDA model was used to extract and analyze topics in review texts. Because the LDA model is a common topic evaluation method that utilizes unsupervised learning, it is considered a relatively mature method for topic analysis (42) for data sets including words, topics, and documents (35, 36). A major characteristic of the LDA model is that it can blend in researchers’ understanding of data with a combination of human experience and machine intelligence. Compared with traditional topic mining methods, the LDA topic model has a useful application effect in analyzing text semantics, which can be used to identify topic information in large-scale document sets or corpora. In contrast to supervised machine learning algorithms, LDA models require less manual input by researchers. The flow chart of the LDA model is presented in Figure 2, and the legend is provided in Table 1.

**Table 1:** LDA model symbol definitions.

Symbol	Definition
θ	Topic distribution of documents
α	Dirichlet distribution parameter of θ
Z	Topic of words in the document
$N_{d}$	All words associated with topic z
*D*	Number of documents
Φ	Word distribution corresponding to each topic
*β*	Dirichlet distribution parameter of Φ
*T*	Number of topics
*W*	Selected words

In the LDA model, the T most relevant top topics are selected to represent topics for all reviews. All reviews correspond to a multinomial distribution of T topics, which is denoted as θ; and α is the Dirichlet distribution parameter of θ. The basic concept of topic word generation under each topic is detailed as follows: each topic is related to a multinomial distribution Φ of $N_{d}$ words in all reviews. *β* is the Dirichlet distribution’s super parameter in the word distribution Φ corresponding to each topic. A topic Z was extracted from the θ distribution of topics corresponding to the comment document. After, a word W was extracted from Φ distribution, corresponding to Topic Z. Repeat this process for Nd times to generate theme words under theme Z.

When extracting topics using the LDA model, the review texts of the input model must be segmented. Word segmentation refers to the splitting of consecutive sentences in review texts into words with smaller granularity without changing the semantics of whole sentences. As with the text mining process in English, Chinese word segmentation also requires that specified stop words (e.g., I, we, of, if, etc.) and some useless characters be removed. Using Python’s Jieba word segmentation package, review texts were divided into words. Jieba word segmentation is a widely used Chinese word segmentation algorithm that combines the Trie tree word graph scanning, dynamic programming, and the Hidden Markov method (43). This algorithm is built on the probability model. By employing word segmentation rules to extract characters, we can divide the given text into a phrase list. To increase the accuracy of word segmentation, the stop vocabulary list from the Harbin Institute of Technology was employed. Moreover, common stop words in medical fields were added to the aforementioned stop vocabulary list, such as “directors,” “doctors,” “hospitals,” and “professors.” Before data were analyzed, characters and words in the designated vocabulary list were filtered out (44).

#### 3.2.2. Topic number

Perplexity is a common indicator for measuring the quality of language models (45). In the LDA model, the optimal review texts topic number K was determined by perplexity. The lower the perplexity value, the better the clustering effect (46). The formula for calculating perplexity is as follows:


$$Perplexity\left(D\right)=\exp\left\{\frac{\overset{N}{\underset{d=1}{\sum }}\log P\left(W_{d}\right)}{\overset{N}{\underset{d=1}{\sum }}N_{d}}\right\}$$

D is the number of test document sets, $W_{d}$ is the lexical sequence of d, and $N_{d}$ is the word quantity of d.

**Figure 3:**
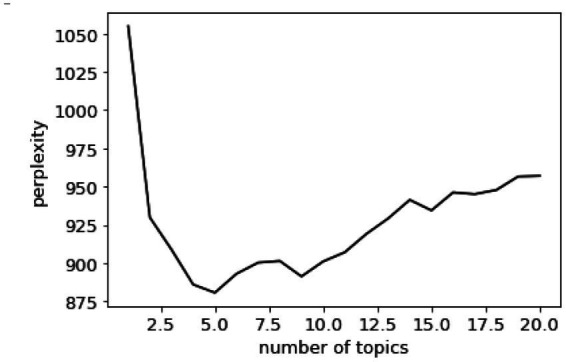
Perplexity value and number of topics.

We determined the number of review topics by perplexity. The calculation results are shown in Figure 3. When the number of topics K = 5, the perplexity value was lowest. Therefore, 5 is the optimal number of topics for online reviews.

#### 3.2.3. Topic–word distribution

After determining the optimal number of topics in the document, we needed to define the distribution of words in each topic. We input review texts after word segmentation and the number of topics into the LDA model to conduct unsupervised training. Then, the LDA model calculated the weight of words in each topic. We reserved words with the top weight as the word distribution of a given topic. Considering the meaning of words in each topic and referencing relevant literature, we manually assigned a name to each topic.

#### 3.2.4. Topic intensity

After defining the distribution of identified topics, we calculated topic intensity, which refers to related topics being mentioned at one time. The stronger the topic intensity, the greater the user attention on that topic in a given period. Topic intensity was calculated based on the review–topic distribution. We obtained the probability of each review text belonging to a topic according to the LDA model and determined the topic probability distribution of different topics for each review. If a topic belongs to a review with high probability, it indicates that reviews are highly related to the topic and will be classified into this topic. After classifying the review texts, we calculated the topic intensity according to the number of reviews corresponding to each topic. The following calculation formula was used:


$$P_{k,t}=\frac{\theta k,t}{Nt}$$

$P_{k,t}$ denotes the intensity of topic *k* at time *t*; $\theta_{k,t}$ is the number of reviews matched by topic *k* at time *t*; and $N_{t}$ is the total number of reviews at time *t*.

### 3.3. Sentiment analysis

ROST sentiment analysis utilizes a sentiment dictionary, which can analyze the proportion of different sentiment sentences in text (47). This study used ROST CM software to analyze the sentiment of user reviews on the platform. ROST CM software is a representative open-source text mining tool developed by Wuhan University. This software is a free social computing platform for Chinese social science research, with features such as mining and analyzing text content information, word segmentation, high-frequency word extraction, cluster analysis, social network, and semantic network analysis (48).

## 4. Results

### 4.1. Topic identification

After data acquisition and processing, LDA model training, and topic extraction, the review texts were categorized according to five topics. We calculated the weight of words associated with each topic based on the LDA model and then determined the topics–words distribution. The result is shown in Table 2. In this study, the five major topics were identified as diagnosis and treatment attitude, medical skills and ethics, treatment scheme, diagnosis effect, and treatment process, which are considered generally representative of the level of user attention to medical services.

**Table 2:** Topic–word distribution.

	Topic	Terms
Topic 1	Diagnosis and treatment attitude	Patience, attitude, scrupulous, detailed, professional, inquires, responsibility, conscientious, careful, solve
Topic 2	Medical skills and ethics	Medical skill, exquisite, medical ethics, attitude, masterly, respectable, brilliant, technology, affable, kindly
Topic 3	Treatment scheme	Treatment, diagnosis, scheme, accuracy, medication, help, confidence, explicit, disease, effect
Topic 4	Diagnosis effect	Operation, treatment, recovery, become better, effect, postoperative, children, success, reexamine, present
Topic 5	Treatment process	Operation, examine, online, hospitalization, register, convenient, outpatient service, make an appointment, conveniently

**Figure 4:**
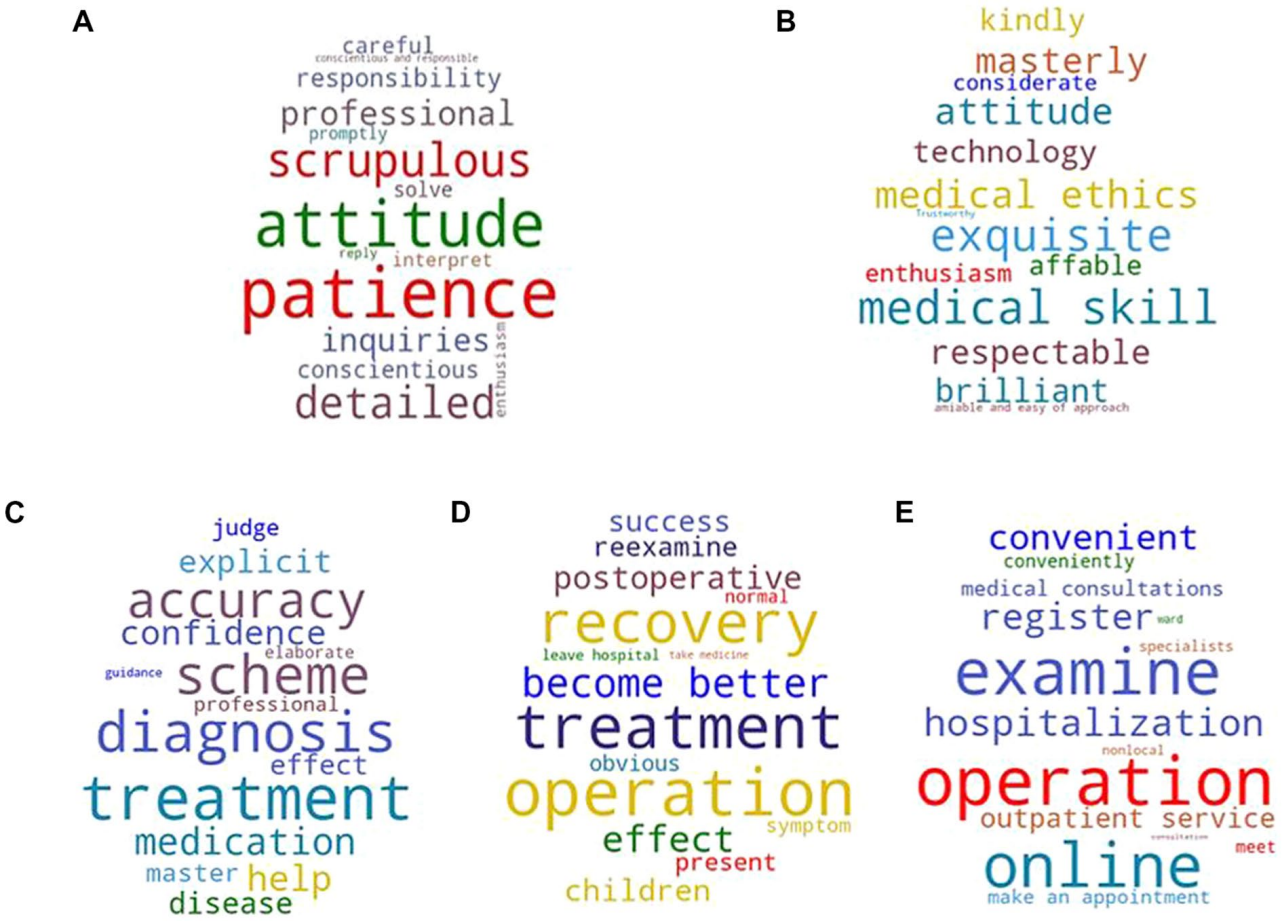
Topic word clouds. **(A)** Topic 1: diagnosis and treatment attitude. **(B)** Topic 2: medical skills and ethics. **(C)** Topic 3: treatment scheme. **(D)** Diagnosis effect. **(E)** Topic 5: treatment process.

Each topic was visualized using a word cloud. The larger the font of a given word, the higher its representative weight, and vice versa. The word clouds are shown in Figure 4. As shown in the word cloud diagram, the keywords of Topic 1, diagnosis and treatment attitude, are patience, attitude, scrupulous, and detailed, which represent doctors’ attitudes and responsibility toward patients. The keywords of Topic 2, medical skill and ethics, include medical skill, exquisite, medical ethics, masterly, and respectable, which represent the level of medical skills and ethics maintained by doctors. The keywords of Topic 3, treatment scheme, involve treatment, diagnosis, scheme, accuracy, and medication, which are related to the disease control plan given by doctors. The keywords of Topic 4, diagnosis effect, include operation, treatment, recovery, become better, and effect, encompassing patients’ recovery experiences after treatment. The keywords of Topic 5, treatment process, include surgery, examination, online, hospitalization, and registration which are associated with how patients interacted with doctors.

We calculated the intensity of each topic, and the results are shown in Table 3. The topic intensity of diagnosis and treatment attitude was highest at 31.428%, and medical ethics was the second highest, with a topic intensity of 25.558%. The topic intensities of diagnosis effect, treatment scheme, and treatment process were relatively low, with 18.654%, 13.664%, and 10.696%, respectively. Topics 1 and 2 were found to have significantly higher topic intensity than the remaining topics, together accounting for over 50% of the topics addressed in all reviews.

**Table 3:** Topic intensity distribution, 2017–2022.

Topic	Topic name	Topic intensity
Topic 1	Diagnosis and treatment attitude	31.428%
Topic 2	Medical skills and ethics	25.558%
Topic 3	Diagnosis effect	18.654%
Topic 4	Treatment scheme	13.664%
Topic 5	Treatment process	10.696%

### 4.2. Topic evolution analysis

Topic evolution refers to changes in topic intensity over time. Regarding the selection and distinction of time periods, this study primarily focused on the development stage of Haodf.com and the change stage in response to COVID-19. Therefore, we selected review texts posted from 2017 to 2022 and distinguished them according to evaluation period.

Due to the outbreak of COVID-19, 2020–2021 is an extraordinary period regarding individuals’ interactions with the healthcare system and industry. In May 2020, the State Council Joint Prevention and Control Mechanism issued guidelines on the work to implement regular pandemic prevention and control. At this time, strategies for preventing and controlling the COVID-19 pandemic changed from emergency to normalization. Considering the time lag of policy implementation, the period of 2020–2021 was divided into two periods based on May 31. Thus, the study period was divided into six stages: 2017, 2018, 2019, 1 January 2020–31 May 2020, 1 June 2020–31 December 2020, and 2021–2022.

**Figure 5:**
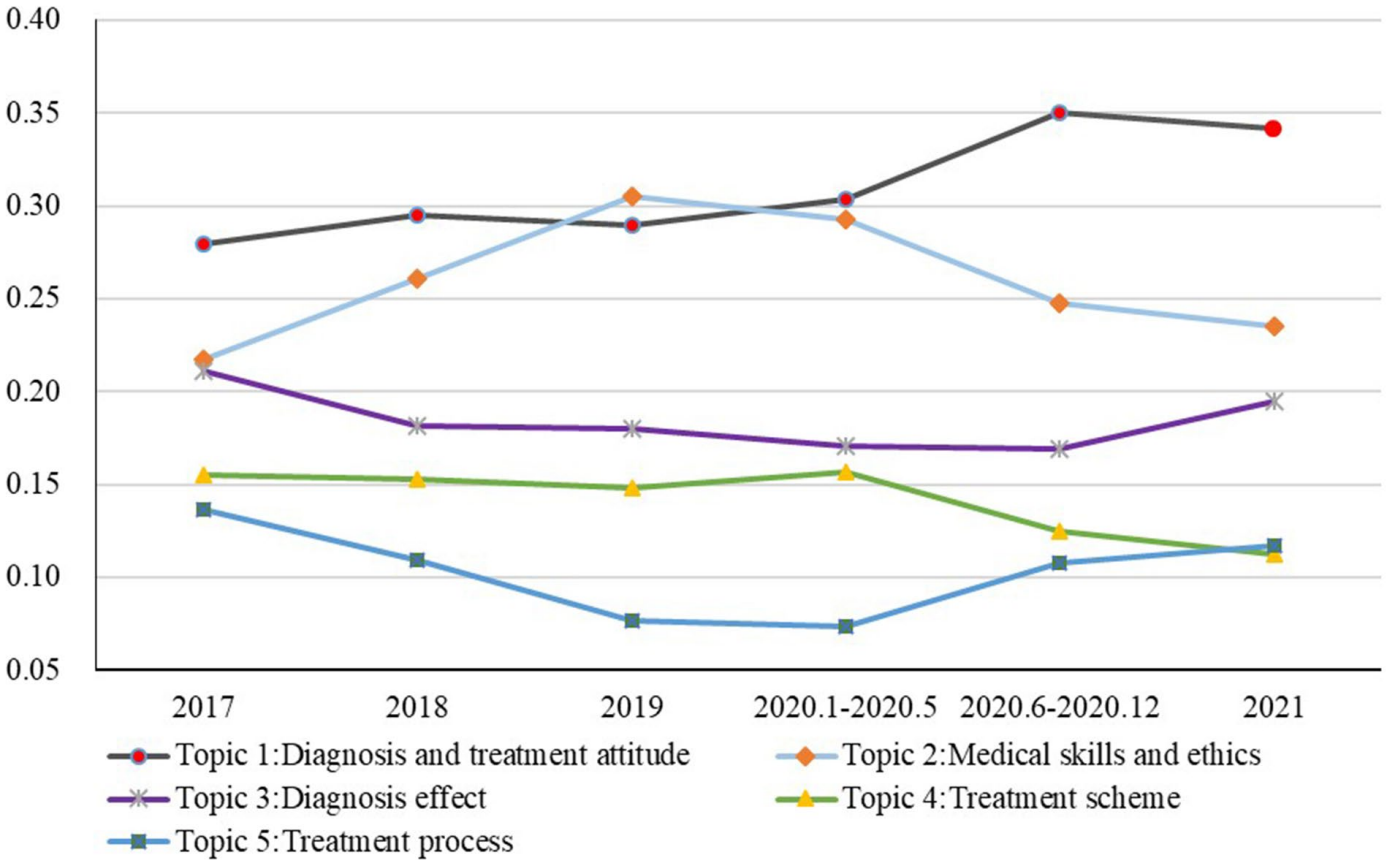
Topic intensity evolution.

We calculated the topic intensity of the six periods and determined the evolution of topic intensity over time. As shown in Figure 5, the horizontal axis represents time, and the vertical axis represents topic intensity. The results are given as a percentage.

First, Topic 1, diagnosis and treatment attitude, was analyzed. The intensity of Topic 1 continued to rise over the study period and remained the highest among all topics. The data shows that from 2017 to 2022, users focused more on the attitude of doctor service during diagnosis and treatment. The intensity of Topic 1 maintained a steady rise before the COVID-19 outbreak, with a continued increase after the outbreak.

Second, we analyzed Topic 2, medical skills and ethics. Topic 2 was found to be the second most important for users. Although the intensity of this topic sharply increased before the COVID-19 outbreak, the intensity showed a downward trend after the COVID-19 outbreak. Especially in 2020, the intensity of Topic 2 declined significantly during the normalization of epidemic prevention and control. A probable reason for this change is that users paid more attention to Topic 1, leading to the decline in the intensity of Topic 2.

Third, we analyzed Topic 3, diagnosis effect. The intensity of Topic 3 was consistently lower than that of Topics 1 and 2 but higher than that of Topics 4 and 5. Even during the COVID-19 outbreak, the intensity of Topic 3 did not fluctuate significantly. In 2020, although the intensity of this topic declined, this change was slight, indicating that interest in the topic may not have been significantly influenced by the pandemic.

Fourth, analyzing Topic 4, treatment scheme, revealed that its intensity was similar to that of Topic 3, which was also relatively stable before the COVID-19 outbreak and decreased slightly after that. Even during the normalization of epidemic prevention and control, the intensity of Topic 4 exhibited a minor decline.

Finally, we analyzed the intensity of Topic 5, treatment process. The intensity of this topic was less than that of the other four topics. It declined moderately before the outbreak of the COVID-19 pandemic and then increased during the normalization of epidemic prevention and control. This trend also indicates that users care more about the treatment process after the COVID-19 outbreak.

In general, Topic 1, diagnosis and treatment attitude, and Topic 2, medical skills and ethics, are the main topics that users focused on, while, after the outbreak of COVID-19, users seem to pay even more attention to the diagnosis and treatment attitude. This trend led to a relative decrease in attention to medical skills and ethics. The trend of Topic 3, diagnosis effect, is similar to Topic 4, treatment scheme. The intensities of these topics are in a relatively stable state. The intensity of Topic 5, treatment process, is relatively minor throughout the period, but it is increasing due to the impact of the COVID-19 pandemic—even surpassing the increase observed in Topic 4.

### 4.3. Sentiment analysis

Review texts also contained information indicating the sentimental tendencies of users, which function as an important indicator of online medical service quality. This section describes the sentiment analysis of review texts obtained from Haodf.com. Based on sentiment analysis, we evaluated user satisfaction regarding online medical services in terms of positive, neutral, and negative sentiments, with positive and negative statements being evaluated in terms of intensity.

When analyzing user reviews, we filtered out characters and expressions that were not recognized. We then imported the processed review data into a .txt file based on the time period assessed. The text was converted to ANSI format for ease of software recognition. Lastly, textual data were imported into ROST CM 6.0 software based on the assessment period. We used the semantic vocabulary of ROST CM to partition semantic sentiment into three types: positive sentiment, neutral sentiment, and negative sentiment. In the present study, positive and negative feeling were further categorized as high, moderate, and normal.

**Figure 6:**
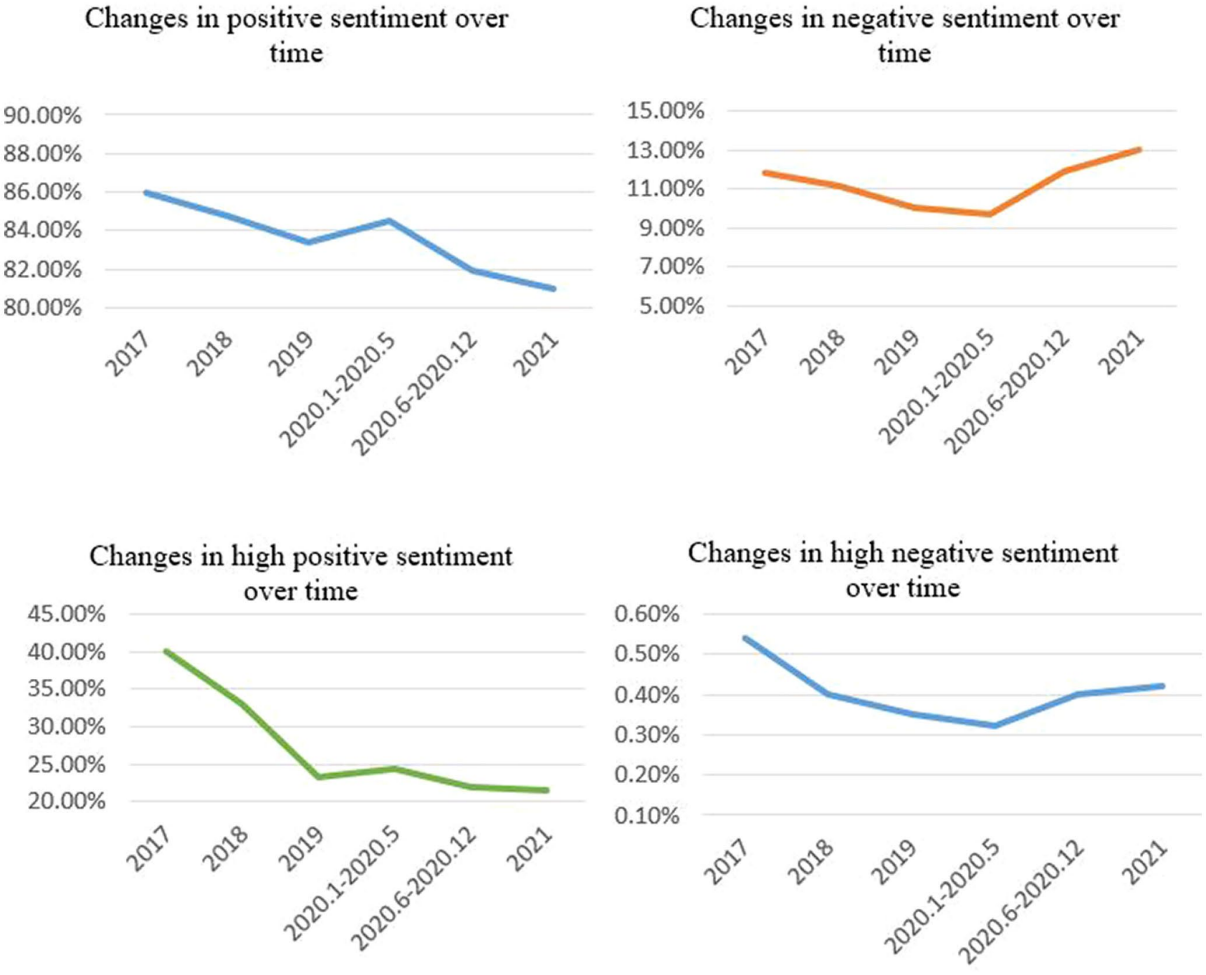
Intensity trends of positive and negative sentiments.

The ROST CM 6.0 software system automatically detected the sentiment of each review to yield sentiment classification results at different stages. The distribution of sentiments from user reviews are presented by evaluation period in Table 4. The intensity trends of positive and negative sentiments are illustrated in Figure 6.

**Table 4:** Sentimental distribution in different stages.

Time	Positive sentiment (%)			Neutral sentiment (%)	Negative sentiment (%)		
2017	Total proportion 85.93			2.28	Total proportion 11.79		
	Normal	Moderate	High		Normal	Moderate	High
	20.71	25.25	39.96		7.91	2.55	0.54
2018	Total proportion 84.77			4.09	Total proportion 11.14		
	Normal	Moderate	High		Normal	Moderate	High
	24.81	26.93	33.03		7.81	2.32	0.40
2019	Total proportion 83.40			6.56	Total proportion 10.04		
	Normal	Moderate	High		Normal	Moderate	High
	30.80	29.43	23.17		7.38	1.80	0.35
2020.1–2020.5	Total proportion 84.48			5.84	Total proportion 9.68		
	Normal	Moderate	High		Normal	Moderate	High
	30.40	29.66	24.42		7.15	1.69	0.32
2020.6–2020.12	Total proportion 81.90			6.15	Total proportion 11.95		
	Normal	Moderate	High		Normal	Moderate	High
	30.93	28.96	22.01		8.78	2.10	0.40
2021–2022	Total proportion 80.97			6.05	Total proportion 12.98		
	Normal	Moderate	High		Normal	Moderate	High
	31.17	28.32	21.48		9.47	2.39	0.42

The sentiment analysis results showed that the positive sentiment distribution remained above 80% in all periods. The positive sentiment data also indicate that most users have good responses to medical experience and a high satisfaction with online medical platforms. In the pre-COVID-19 stage, the overall positive sentiment showed a weak downward trend, while the neutral sentiment showed a weak upward trend. The high positive sentiment also showed a weak downward trend, indicating that user satisfaction slightly decreased before COVID-19 and implied that users have higher requirements for medical services.

During the emergency period of the COVID-19 pandemic (namely, the first half of 2020), positive sentiment increased, exceeding that in 2018 and 2019. In the second half of 2020, compared with other periods, positive sentiment decreased significantly. On the contrary, the negative sentiment rose slightly. In 2021, positive sentiment maintained a slight downward trend, which may have occurred as the demand for medical services increased due to the pandemic’s impacts. Another reason may be that during the pandemic, medical resources were strained, and medical service quality declined to some extent, which could lead to negative sentiment. For example, during the outbreak of COVID-19, the number of online medical users significantly increased compared to that during the non-pandemic period, which made it difficult for doctors to cope with the sudden surge of users. This factor may have led to negative sentiment in users, thus impacting the sentiment distribution of review texts online.

## 5. Discussion

### 5.1. Research conclusions

In this study, we used Python to crawl user review data from Haodf.com, which is a widely known online medical consulting platform in China. After data cleaning, we obtained 323,519 review texts from 2017 to 2022. Then, using an LDA model and ROST CM software, key topics were identified, and sentiment analysis was conducted. Finally, we explored user focus change trends. Our research obtains the following significant key findings.

Firstly, this research found that patients placed a high value on doctors’ service and technical quality. Data Mining of the Reviews shows that users pay the most attention to doctors’ attitudes toward diagnosis and treatment as well as medical skills and ethics.

Similar results have been proposed by Ko et al. (49) have proposed similar findings. They found that patients pay attention not only to the technical quality of physicians, but also to the quality of the service. Two critical aspects of service quality in medicine are technical quality and patient experience. Technical quality and patient experience are two critical aspects of medical Service quality. Furthermore, this result is in agreement with those proposed by Yang et al. (50), who found that medical skills and service feedback were the main factors affecting patients’ selection of a doctor for consultation. Similarly, Chen et al. (6) conclude that informational and emotional support are two main types of social support provided by physicians. Patients value emotional support from the doctor more than informational support.

One possible explanation is that doctors’ professional ability and attitudes toward services are substitutable from the patients point of view (50). Physicians and patients may need assistance with face-to-face communication in the online consultation, which will result in insufficient interactions between them (16, 51). Because there is an asymmetry of professional information between physicians and patients, patients cannot judge physicians’ professional abilities (11). Doctors’ behavior such as patiently listening, responding quickly, explaining things carefully is an observable signal of service quality. More importantly, the behavior may reduce patients’ anxiety, causing them to perceive politeness and respect from doctors (3, 18, 52). Therefore, not only do patients pay attention to the professional competence of doctors, they also pay attention to the attitude of doctors.

Secondly, our findings suggest that patients are more concerned about service quality following the COVID-19 outbreak. In contrast to previous research that has shown patients to be concerned about physicians’ service attitudes (3, 10, 12), it did not analyze the impact of COVID-19 on satisfaction. Our research examines the topics and sentimental change rules of user review texts during the COVID-19 outbreak. In our research, we examine the sentiment-changing topics and rules of user review texts during the COVID-19 outbreak. We found that users continued to focus on physicians’ attitudes toward diagnosis and treatment, while attention to medical skills and ethics declined somewhat. Users’ interest in these topics fluctuated widely due to the COVID-19 pandemic. User attention to diagnostic effects, treatment regimens, and treatment processes also changed during the pandemic.

Our findings reveal that the emotional support provided by physicians is needed for patients during the COVID-19 pandemic. The public is required to maintain social distancing in order to contain the epidemic. Because of these restrictions, people have experienced physical anxiety, which may have led to psychological illnesses such as depression, episodes, insomnia, and the like (53–55). When physicians resolve patients’ emotional demands, patients need emotional reassurance from physicians to alleviate psychological stress and improve treatment (16), such as depression and anxiety, in a conscientious manner. These can positively influence patients level of satisfaction (23, 56). Doctors’ soothing and reassurance is of the essence in this situation (6). For example, patients will pay significantly more attention to service quality and emotional support during an online consultation.

Third, from the perspective of user sentiments, the results suggest that most reviews indicated a positive attitude toward online medical platforms. This study aligns with previous research that online medical services have gained widespread acceptance (57–61). This could be due to the fact that online medical platforms can offer patients convenient and effective clinical care. Patients can seek professional advice from physicians via online medical platforms without regard to time or space constraints. Patients can also provide feedback on the physicians’ service attitudes and expertise (3). As a result, physician reviews reflect physicians’ attitudes and service quality, which can assist patients in making decisions and selecting physicians (9). Physicians’ publicly available information can reduce patients’ psychological risk perceptions (11). Another possible explanation is that government regulation is effective. Following 2017, the Chinese government implemented a number of significant policies and regulations in the emerging online healthcare market. The standard development stage has been reached for China’s online medical platform. As a result, our findings indicate that users are pleased with their platform experience.

Finally, we found that during the normalization of epidemic prevention and control following the COVID-19 outbreak, user experiences regarding the quality of medical services began declining. This special finding can be attributed to a marked negative impact of the COVID-19 pandemic on the physical and mental health of various affected people (54, 62). Aside from that, physicians were bombarded with far more patients than in the past. To alleviate stress and health anxiety, patients require more emotional support, such as being genuinely cared for and respected, as well as good communication (63). Patients will have higher expectations for service quality in this situation than usual. Many patients, on the other hand, prefer face-to-face communication. Because of the widespread spread of the COVID-19 pandemic, there is no other option but to seek medical advice online (64). According to Ren and Ma (52), 1.11 billion people visited the Haodf.com website during the pandemic. In a short period of time, the number of online medical consultations skyrockets. Doctors struggle to keep up with the volume of online consultations. As a result, many patients’ health demands were not met. It is also difficult to guarantee service quality. As previously discussed, it is natural that some patients rated their online consultations as less than satisfactory.

### 5.2. Theoretical contributions

The main contributions of this study are as follows: first, we extract the service features by using physician reviews to determine the patients’ concerns. Although the prior literature has elaborated on several factors in online medical services, those studies mainly focused on doctors, patients, platforms, etc. (11, 12, 18). We investigate the evolution of platform service quality over time, focusing on the impact of the epidemic. Using a temporal analysis approach, our research reports on the evolution trends of major topics of concern for Chinese users of online medical service platforms. Our conclusions are objective, comprehensive, and well-developed. These findings can be used to assess changes in user demand for online medical services.

Second, we investigated users’ evaluation of the service quality of a major online medical platform via sentiment analysis. This research can help comprehensively evaluate online medical platforms’ quality in China. Some studies use questionnaires to investigate patient satisfaction with online medical platforms (64). Although some studies have rated the service quality of online medical platforms (65), emotional analysis methods are rarely used to rate the platform in China (3). Using sentiment analysis, we can rate user satisfaction with online medical services. Our findings provide insights into patients’ online emotional needs.

Third, this study investigated evolutions in topics and sentiment from user reviews on online medical platforms before and after the onset of the COVID-19 pandemic, which provides data support for understanding the impact of the COVID-19 pandemic on online medical services.

### 5.3. Practical significance

This study reports the change characteristics of user needs and experiences in engaging with online medical services, especially during the COVID-19 pandemic. These results provide a reference for understanding how users select medical treatments or practitioners and support online medical platforms and doctors in making decisions. Users tended to emphasize diagnosis and treatment attitude along with medical skills and ethics. Therefore, the attitudes of practitioners during online diagnosis and treatment should be considered in practice. Moreover, according to user preferences, doctors should improve their service attitude, skills, and serviceability consistently. For example, when a doctor conducts an online consultation, they should guarantee timely responsiveness as well as an emotionally supportive attitude. Doctors must reply to users’ consults accurately and with great detail, to ensure users feel the doctors’ positive service attitude.

For online medical platforms, a reward system should be established to improve service attitudes and cultivate medical skills and ethics by doctors using the platform. For example, platforms can implement a scoring system to recommend doctors with good medical skills and ethics to users. The platform can improve the ranking of good doctors through recommendation systems, making it easier for users to choose suitable doctors. Moreover, in the context of the COVID-19 pandemic, hospitals should develop multi-channel diagnosis and treatment methods, combining online and offline approaches to facilitate an effective and efficient diagnosis and treatment process.

### 5.4. Research limitations and future research

Our study was subject to some limitations. First, although Haodf.com is one of the leading Internet medical platforms in China, collecting data from only one site necessarily limits the generalizability of our findings. The research conclusions may be different for data from other platforms. Subsequent studies should evaluate multiple online medical platforms. The conclusion of this study may have another limitation: the calculated emotion result can only reflect the user’s emotion and may have some deviation from the platform’s service quality. We hope to find a more appropriate method of evaluating service quality in the future. Finally, this study did not consider how differences in illness severity result in different patient needs. Patients’ illness severity is an important characteristic. As a result, patients with different illnesses may be more or less sensitive to physicians’ professional ability and service attitudes. The effects of illness severity should be considered in future research.

## 6. Conclusion

The online medical platform is a different way to manage health and provide patient consultations. During the COVID-19 epidemic, online medical consultation platforms effectively solved the problem of medical shortage. The information, such as doctors’ professional skills and service attitude, provides an important basis for patients’ decisions. We identify the topic and analyze user sentiments from review texts on Haodf.com. The results showed that users mainly focused on diagnosis, treatment, medical skills and ethics. After the appearance of COVID-19, users’ positive sentiments on the platform decreased significantly. These findings contribute to online healthcare market research and practice.

## Data availability statement

Publicly available datasets were analyzed in this study. This data can be found at: https://www.haodf.com/.

## Author contributions

CL, JY, JW, and SL conceived the idea of the manuscript, designed the research, collected and analyzed the data, and wrote the manuscript. CL, JW, and YL modified the manuscript. All authors contributed to the article and approved the submitted version.

## Funding

This study was supported by the National Social Science Fund of China (grant no. 21BGL238), the Cultivation Programmer for Young Backbone Teachers in Colleges and Universities in Henan Province (grant no. 2020GGJS087), the Cultivation Programmer for Young Backbone Teachers in Henan University of technology (grant no. 21420127), the Innovation Training Program for College Students in Henan Province (grant no. 202210463048), the Research and Practice Project of Undergraduate Education and Teaching Reform in Henan University of Technology (grant no. JXYJ2021021), the Annual Project of Henan Provincial Philosophy and Social Science Planning (grant nos. 2022CSH033 and 2022BJJ107), the Henan Soft Science Research Plan Project (grant no. 232400412044), and the High level talent fund project of Henan University of Technology (grant no. 2019SBS007).

## Conflict of interest

The authors declare that the research was conducted in the absence of any commercial or financial relationships that could be construed as a potential conflict of interest.

## Publisher’s note

All claims expressed in this article are solely those of the authors and do not necessarily represent those of their affiliated organizations, or those of the publisher, the editors and the reviewers. Any product that may be evaluated in this article, or claim that may be made by its manufacturer, is not guaranteed or endorsed by the publisher.
