# Supplementary material for: Topic evolution and sentiment comparison of user reviews on an online medical platform in response to COVID-19: taking review data of Haodf.com as an example
Source: Front Public Health. 2023 Jun 2;11:1088119. doi: 10.3389/fpubh.2023.1088119 (PMC10272356; doi:10.3389/fpubh.2023.1088119)
Supplement: Supplementary file 1 [file Data_Sheet_1.DOCX]

**Appendix I**

**Code for reviews crawling**

import requests

from pyquery import PyQuery

import csv

**爬取疾病链接（Crawl disease link）**

#新建csv

csvf = open('Desktop/csv文件/疾病链接.csv', 'a+', encoding='utf-8', newline='')

fieldnames = ['疾病名称','url1']

writer = csv.DictWriter(csvf, fieldnames=fieldnames)

writer.writeheader()

url = 'https://www.haodf.com/citiao/list-jibing.html'

headers = {'user-agent': 'Mozilla/5.0 (Windows NT 10.0; Win64; x64) AppleWebKit/537.36 (KHTML, like Gecko) Chrome/99.0.4844.51 Safari/537.36'}

resp = requests.get(url, headers=headers)

doc = PyQuery(resp.text)

#解析&存储

for url1 in doc.items('.topli'):

疾病名称 = url1('.topli').text()

url1 = url1('.topli a').attr('href')

data = {'疾病名称': 疾病名称,

'url1': ' https://www.haodf.com'+url1

}

writer.writerow(data)

#关闭csv

csvf.close()

import pandas as pd

df = pd.read_csv('Desktop/csv文件/疾病链接.csv')

df

**爬取医生链接（Crawl doctor link）**

import requests

from pyquery import PyQuery

import csv

import pandas as pd

#新建csv

csvf = open('C:/Users/lenovo/Desktop/csv文件/医生链接.csv', 'a+', encoding='utf-8', newline='')

fieldnames = ['姓名','级别','学位','所属医院科室','擅长','问诊价格','票数','疗效满意度','推荐热度','主治疾病','内层url']

writer = csv.DictWriter(csvf, fieldnames=fieldnames)

writer.writeheader()

headers = {'user-agent': 'Mozilla/5.0 (Windows NT 10.0; Win64; x64) AppleWebKit/537.36 (KHTML, like Gecko) Chrome/99.0.4844.51 Safari/537.36'}

for i in range(len(df)):

c = df['疾病名称'][i]

template = df['url1'][i]+'?p={page}'

for p in range(1, 8):

# print(p)

url3 = template.format(page=p)

resp = requests.get(url3, headers=headers)

doc = PyQuery(resp.text)

#解析&存储

for k in doc.items('.item '):

内层url = k('.item-bd').attr('href')

姓名 = k('.name').text()

级别 = k('.grade').text()

学位 = k('.edu-grade').text()

所属医院科室 = k('.hos-faculty').text()

擅长 = k('.goodat').text()

问诊价格 = k('.p-i-orange').text()

票数 = k('.disease-label ').text()

疗效满意度 = k('.percent').text()

推荐热度 = k('.score').text()

try:

if "hospital" not in 内层url:

data = { '姓名': 姓名,

'级别': 级别,

'学位': 学位,

'所属医院科室': 所属医院科室,

'擅长': 擅长,

'问诊价格': 问诊价格,

'票数': 票数,

'疗效满意度': 疗效满意度,

'推荐热度': 推荐热度,

'主治疾病': c,

'内层url':内层url}

writer.writerow(data)

except:

None

#关闭csv

csvf.close()

import pandas as pd

df7 = pd.read_csv('Desktop/csv文件/医生链接.csv')

df7

c=df7.drop_duplicates('姓名',keep='first')

c

df2=c.reset_index()

df2

**爬取评论文本（Crawl reviews text）**

import requests

from pyquery import PyQuery

import csv

import re

#新建csv

csvf = open('Desktop/csv文件/近期评价数据.csv', 'a+', encoding='utf-8', newline='')

fieldnames = ['医生姓名','患者姓名','病症','时间','诊疗情况','评价标签','评价']

writer = csv.DictWriter(csvf, fieldnames=fieldnames)

writer.writeheader()

headers = {'user-agent': 'Mozilla/5.0 (Windows NT 10.0; Win64; x64) AppleWebKit/537.36 (KHTML, like Gecko) Chrome/99.0.4844.51 Safari/537.36'}

for r in range(len(df2)):

b = df2['姓名'][r]

pattern = '.html'

repl = ''

url=re.sub(pattern, repl,df2['内层url'][r])

template = url+'/pingjia-zhenliao.html?siftKey=1&p={page}'

for p in range(1, 51):

url = template.format(page=p)

resp = requests.get(url, headers=headers)

doc = PyQuery(resp.text)

#解析&存储

for ping in doc.items('.list-item'):

患者姓名 = ping('.patient-name').text()

病症 = ping('.disease-tag').text()

时间 = ping('.date').text()

诊疗情况 = ping('.summary-group').text()

评价标签 = ping('.trait-bd').text()

评价 = ping('.content').text()

data2 = {'医生姓名':b,

'患者姓名': 患者姓名,

'病症': 病症,

'时间': 时间,

'诊疗情况':诊疗情况,

'评价标签':评价标签,

'评价': 评价,

}

writer.writerow(data2)

# print(data)

# break

#关闭csv

csvf.close()
