# Supplementary material for: Topic evolution and sentiment comparison of user reviews on an online medical platform in response to COVID-19: taking review data of Haodf.com as an example
Source: Front Public Health. 2023 Jun 2;11:1088119. doi: 10.3389/fpubh.2023.1088119 (PMC10272356; doi:10.3389/fpubh.2023.1088119)
Supplement: Supplementary file 2 [file Data_Sheet_2.DOCX]

**Appendix II**

**Code for Perplexity**

from sklearn.feature_extraction.text import TfidfVectorizer, CountVectorizer

from sklearn.decomposition import LatentDirichletAllocation

import re

import jieba

import matplotlib.pyplot as plt

import pandas as pd

df2 = pd.read_excel('Desktop/csv文件/excel文件/整体数据.xlsx')

df2

stoptext = open('Desktop/csv文件/中文停用词表.txt', encoding='utf-8').read()

stopwords = stoptext.split('\n')

def clean_text(text):

words = jieba.lcut(text)

words = [w for w in words if w not in stopwords]

return ' '.join(words)

df2['content2'] = df2['评价'].apply(clean_text)

df2.head()

vv=df2['content2'].dropna()

#n_features = 1000

tf_vectorizer = CountVectorizer(strip_accents = 'unicode',

# max_features=n_features,

stop_words='english',

max_df = 0.5,

min_df = 20)

tf2 = tf_vectorizer.fit_transform(vv)

plexs = []

scores = []

n_max_topics =21

for i in range(1,n_max_topics):

print(i)

lda = LatentDirichletAllocation(n_components=i, max_iter=100,#迭代次数

learning_method='batch',

learning_offset=50,random_state=100)

lda.fit(tf2)

plexs.append(lda.perplexity(tf2))

scores.append(lda.score(tf2))

n_t=20#区间最右侧的值。注意：不能大于n_max_topics

x=list(range(1,n_t+1))

plt.plot(x,plexs[0:n_t],color='black')

plt.xlabel("number of topics")

plt.ylabel("perplexity")

plt.rcParams['font.size']=10

# plt.savefig('C:\代码')#保存图片

plt.show()


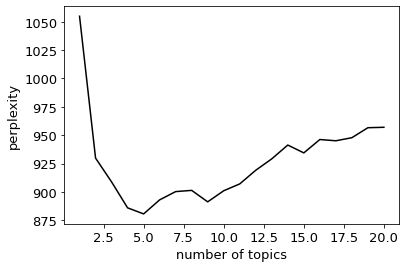


**运行结果（Running results）**
