# Supplementary material for: Topic evolution and sentiment comparison of user reviews on an online medical platform in response to COVID-19: taking review data of Haodf.com as an example
Source: Front Public Health. 2023 Jun 2;11:1088119. doi: 10.3389/fpubh.2023.1088119 (PMC10272356; doi:10.3389/fpubh.2023.1088119)
Supplement: Supplementary file 3 [file Data_Sheet_3.DOCX]

**Appendix III**

**Code for Topic identify and topic classification**

import pandas as pd

df = pd.read_excel('./csv文件/excel文件/整体数据.xlsx')

df

import re

import jieba

stoptext = open('./csv文件/中文.txt', encoding='utf-8').read()

stopwords = stoptext.split('\n')

def clean_text(text):

words = jieba.lcut(text)

words = [w for w in words if w not in stopwords]

return ' '.join(words)

df['content'] = df['评价'].apply(clean_text)

df.head()

from sklearn.feature_extraction.text import CountVectorizer,TfidfVectorizer

vectorizer = CountVectorizer(max_df=0.5,

min_df=20)

doc_term_matrix = vectorizer.fit_transform(df['content'])

doc_term_matrix

from sklearn.decomposition import LatentDirichletAllocation

# 构建LDA话题模型

lda_model = LatentDirichletAllocation(n_components=5) # 话题数

lda_output = lda_model.fit_transform(doc_term_matrix)

print(lda_model) #模型参数

print(lda_output) #话题分布情况

vectorizer.get_feature_names()

def show_topics(vectorizer, lda_model, top_n=20):

import numpy as np

keywords = np.array(vectorizer.get_feature_names())

topic_keywords = []

#话题-词语权重矩阵

for topic_weights in lda_model.components_:

#获得权重最大的top_n词语的权重向量

top_keyword_locs = (-topic_weights).argsort()[:top_n]

#在keywords中找到对于的关键词

topic_keywords.append(keywords.take(top_keyword_locs))

return topic_keywords

topic_keywords = show_topics(vectorizer= vectorizer,

lda_model = lda_model,

top_n = 20)

df_topic_keywords = pd.DataFrame(topic_keywords)

df_topic_keywords.columns = ['Word-'+str(i) for i in range(df_topic_keywords.shape[1])]

df_topic_keywords.index = ['Topic-'+str(i) for i in range(df_topic_keywords.shape[0])]

df_topic_keywords


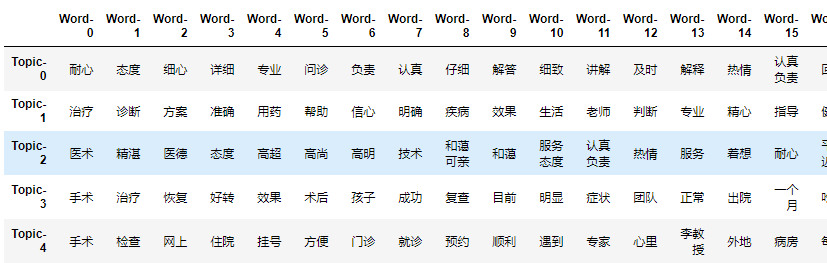


**Topic Extraction Run Result**

import numpy as np

# cntVector = CountVectorizer()

# cntTf = cntVector.fit_transform(cmtlst)

# 构建文档-词频矩阵

lda_output = lda_model.transform(doc_term_matrix)

# 列名

topicnames = ["Topic" + str(i)

for i in range(lda_model.n_components)]

docnames = ["Doc" + str(i)

for i in range(len(df['评价']))]

# 转化为pd.DataFrame

df_document_topic = pd.DataFrame(np.round(lda_output, 2),

columns=topicnames,

index=docnames)

# Get dominant topic for each document

dominant_topic = np.argmax(df_document_topic.values, axis=1)

df_document_topic['dominant_topic'] = dominant_topic

# Styling

def color_green(val):

color = 'green' if val > .1 else 'black'

return 'color: {col}'.format(col=color)

def make_bold(val):

weight = 700 if val > .1 else 400

return 'font-weight: {weight}'.format(weight=weight)

# Apply Style

df_document_topics = df_document_topic.sample(10).style.applymap(color_green).applymap(make_bold)

df_document_topics


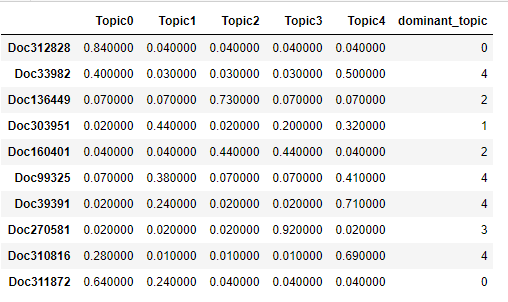


**Reviewss-topics classification Run Result**

df_topic_distribution = df_document_topic['dominant_topic'].value_counts().reset_index(name="Num Documents")

df_topic_distribution.columns = ['Topic Num', 'Num Documents']

df_topic_distribution

df_document_topic.reset_index(drop=False,inplace=True)

df_document_topic

cc=pd.concat([df_document_topic,df], ignore_index=True,axis=1)

cc.to_excel('./csv文件/excel文件/预测数据.xlsx') #合并保存
