# Supplementary material for: Topic evolution and sentiment comparison of user reviews on an online medical platform in response to COVID-19: taking review data of Haodf.com as an example
Source: Front Public Health. 2023 Jun 2;11:1088119. doi: 10.3389/fpubh.2023.1088119 (PMC10272356; doi:10.3389/fpubh.2023.1088119)
Supplement: Supplementary file 4 [file Data_Sheet_4.DOCX]

**Appendix IV**

**Code for word cloud**

import jieba

import numpy as np # numpy数据处理库

import wordcloud # 词云展示库

from PIL import Image # 图像处理库

import matplotlib.pyplot as plt # 图像展示库

from matplotlib import colors

kk=[('patience', 15), ('attitude', 14), ('scrupulous',13), ('detailed', 12), ('professional', 11), ('inquiries', 10), ('responsibility', 9), ('conscientious', 8), ('careful', 7), ('solve', 6), ('interpret', 5), ('promptly', 4), ('enthusiasm',3), ('reply', 2), ('conscientious and responsible',1)]

import numpy

from matplotlib import colors

color_list =['red','blue','green','#3d9cdf','#cd6136','#1685a9','#4b5cc4','#8c4356','#d9b611','#815476']

mask = np.array(Image.open('Desktop/论文文件数据/csv文件/01e54b5de9be88a801213853aeb0be.jpg@1280w_1l_2o_100sh.jpg')) # 导入云图背景图片

wc = wordcloud.WordCloud(

# font_path='./data/STXINGKA.TTF', # 设置字体格式

background_color="white",#背景颜色

mask=mask, # 设置背景图片

#max_words=500, # 最多显示词数

max_font_size=300, # 字体最大值

min_font_size=10, #字体最小值

colormap=colors.ListedColormap(color_list),

width=1000,

height=800,

)

words = dict(kk)

wc.generate_from_frequencies(words) # 从字典生成词云

image_colors = wordcloud.ImageColorGenerator(mask) # 从背景图建立颜色方案

plt.figure(figsize=(14,8))

plt.rcParams['figure.dpi'] = 100 # 修改dpi

plt.imshow(wc) # 显示词云

plt.axis('off') # 关闭坐标轴

# plt.savefig('./中文评论词云图.png')#保存图片

plt.show() # 显示图像


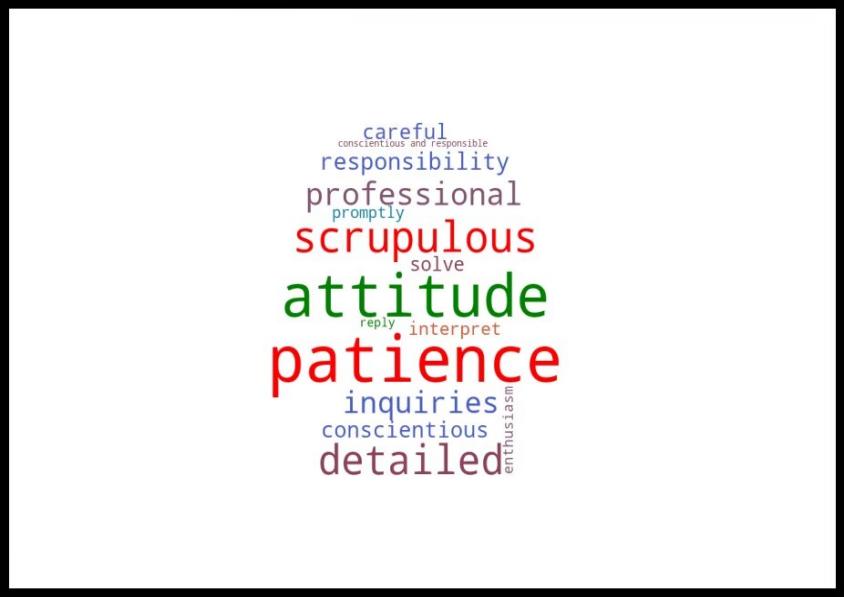


**Running result**

dd=[('medical skill', 15), ('exquisite', 14), ('medical ethics',13), ('attitude', 12), ('masterly', 11), ('respectable', 10), ('brilliant', 9), ('technology', 8), ('kindly', 7), ('affable', 6), ('enthusiasm', 5), ('considerate', 4), ('amiable and easy of approach',3), ('Trustworthy', 2), ('good',1)]

import numpy

from matplotlib import colors

color_list = ['red','blue','green','#3d9cdf','#cd6136','#1685a9','#4b5cc4','#8c4356','#d9b611','#815476']

mask = np.array(Image.open('Desktop/论文文件数据/csv文件/01e54b5de9be88a801213853aeb0be.jpg@1280w_1l_2o_100sh.jpg')) # 导入云图背景图片

wc = wordcloud.WordCloud(

# font_path='./data/STXINGKA.TTF', # 设置字体格式

background_color="white",#背景颜色

mask=mask, # 设置背景图片

#max_words=500, # 最多显示词数

max_font_size=450, # 字体最大值

min_font_size=10, #字体最小值

colormap=colors.ListedColormap(color_list),

width=1000,

height=800,

)

words = dict(dd)

wc.generate_from_frequencies(words) # 从字典生成词云

image_colors = wordcloud.ImageColorGenerator(mask) # 从背景图建立颜色方案

plt.figure(figsize=(14,8))

plt.rcParams['figure.dpi'] = 100 # 修改dpi

plt.imshow(wc) # 显示词云

plt.axis('off') # 关闭坐标轴

# plt.savefig('./中文评论词云图.png')#保存图片

plt.show() # 显示图像


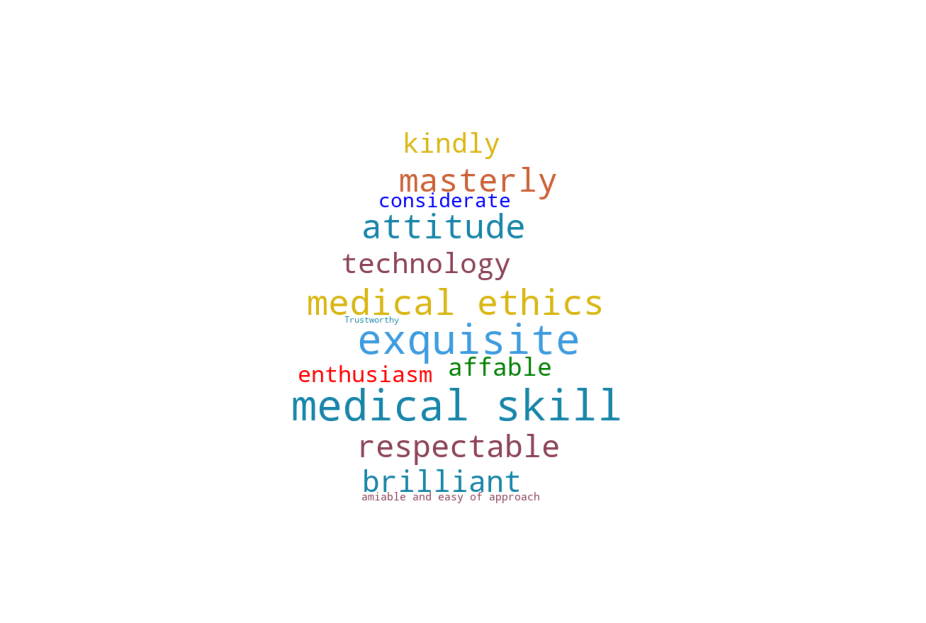


**Running result**

bb=[('treatment', 15), ('diagnosis', 14), ('scheme',13), ('accuracy', 12), ('medication', 11), ('help', 10), ('confidence', 9), ('explicit', 8), ('disease', 7), ('effect', 6), ('master', 5), ('judge', 4), ('professional',3), ('elaborate', 2), ('guidance',1)]

import numpy

from matplotlib import colors

color_list = ['red','blue','green','#3d9cdf','#cd6136','#1685a9','#4b5cc4','#8c4356','#d9b611','#815476']

mask = np.array(Image.open('Desktop/论文文件数据/csv文件/01e54b5de9be88a801213853aeb0be.jpg@1280w_1l_2o_100sh.jpg')) # 导入云图背景图片

wc = wordcloud.WordCloud(

# font_path='./data/STXINGKA.TTF', # 设置字体格式

background_color="white",#背景颜色

mask=mask, # 设置背景图片

#max_words=500, # 最多显示词数

max_font_size=400, # 字体最大值

min_font_size=10, #字体最小值

colormap=colors.ListedColormap(color_list),

width=1500,

height=1000,

)

words = dict(bb)

wc.generate_from_frequencies(words) # 从字典生成词云

image_colors = wordcloud.ImageColorGenerator(mask) # 从背景图建立颜色方案

plt.figure(figsize=(14,8))

plt.rcParams['figure.dpi'] = 100 # 修改dpi

plt.imshow(wc) # 显示词云

plt.axis('off') # 关闭坐标轴

# plt.savefig('./中文评论词云图.png')#保存图片

plt.show() # 显示图像


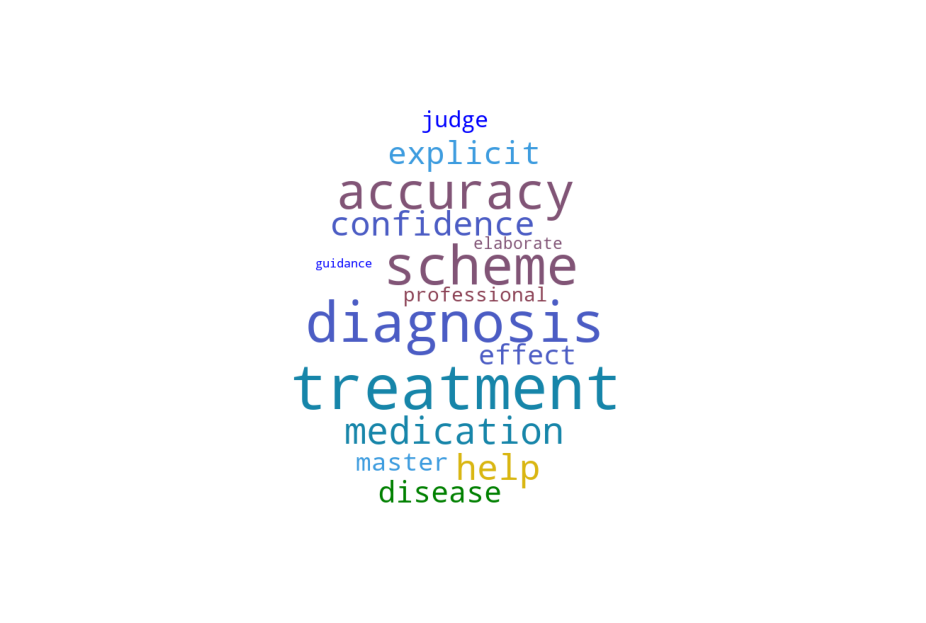


**Running result**

tt=[('operation', 15), ('treatment', 14), ('recovery',13), ('become better', 12), ('effect', 11), ('postoperative', 10), ('children', 9), ('success', 8), ('reexamine', 7), ('present', 6), ('obvious', 5), ('symptom', 4), ('normal',3), ('leave hospital', 2), ('take medicine',1)]

import numpy

from matplotlib import colors

color_list = ['red','blue','green','#3d9cdf','#cd6136','#1685a9','#4b5cc4','#8c4356','#d9b611','#815476','#3b2e7e']

mask = np.array(Image.open('Desktop/论文文件数据/csv文件/01e54b5de9be88a801213853aeb0be.jpg@1280w_1l_2o_100sh.jpg')) # 导入云图背景图片

wc = wordcloud.WordCloud(

# font_path='./data/STXINGKA.TTF', # 设置字体格式

background_color="white",#背景颜色

mask=mask, # 设置背景图片

#max_words=500, # 最多显示词数

max_font_size=400, # 字体最大值

min_font_size=10, #字体最小值

colormap=colors.ListedColormap(color_list),

width=1500,

height=1000,

)

words = dict(tt)

wc.generate_from_frequencies(words) # 从字典生成词云

image_colors = wordcloud.ImageColorGenerator(mask) # 从背景图建立颜色方案

plt.figure(figsize=(14,8))

plt.rcParams['figure.dpi'] = 100 # 修改dpi

plt.imshow(wc) # 显示词云

plt.axis('off') # 关闭坐标轴

# plt.savefig('./中文评论词云图.png')#保存图片

plt.show() # 显示图像


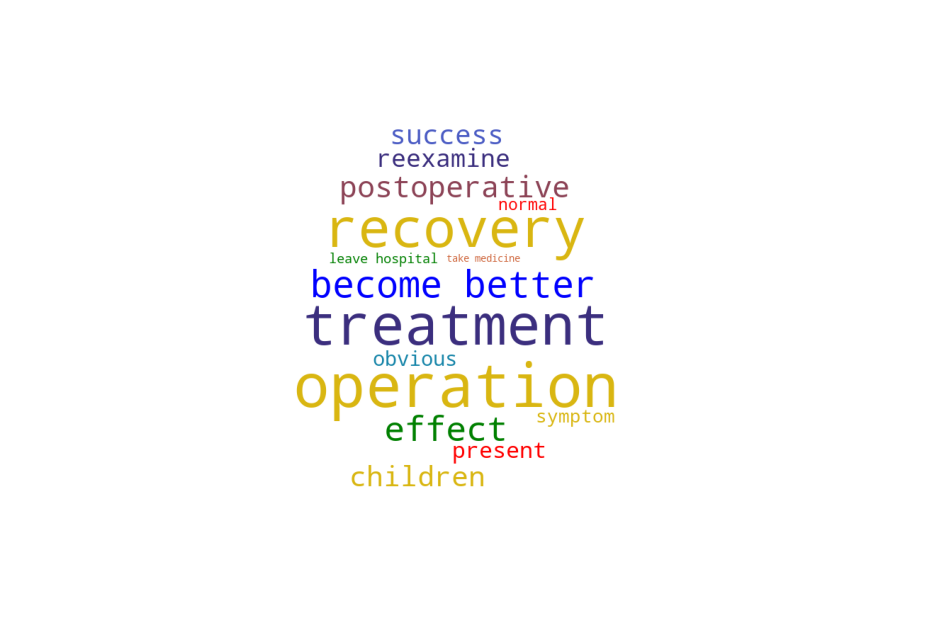


**Running result**

a=[('operation', 15), ('examine', 14), ('online',13), ('hospitalization', 12), ('register', 11), ('convenient', 10), ('outpatient service', 9), ('medical consultations', 8), ('make an appointment', 7), ('conveniently', 6), ('meet', 5), ('specialists', 4), ('nonlocal',3), ('ward', 2), ('consultation',1)]

import numpy

from matplotlib import colors

color_list = ['red','blue','green','#3d9cdf','#cd6136','#1685a9','#4b5cc4','#8c4356','#d9b611','#815476']

mask = np.array(Image.open('Desktop/论文文件数据/csv文件/01e54b5de9be88a801213853aeb0be.jpg@1280w_1l_2o_100sh.jpg')) # 导入云图背景图片

wc = wordcloud.WordCloud(

# font_path='./data/STXINGKA.TTF', # 设置字体格式

background_color="white",#背景颜色

mask=mask, # 设置背景图片

#max_words=500, # 最多显示词数

max_font_size=400, # 字体最大值

min_font_size=10, #字体最小值

colormap=colors.ListedColormap(color_list),

width=1500,

height=1000,

)

words = dict(a)

wc.generate_from_frequencies(words) # 从字典生成词云

image_colors = wordcloud.ImageColorGenerator(mask) # 从背景图建立颜色方案

plt.figure(figsize=(14,8))

plt.rcParams['figure.dpi'] = 100 # 修改dpi

plt.imshow(wc) # 显示词云

plt.axis('off') # 关闭坐标轴

# plt.savefig('./中文评论词云图.png')#保存图片

plt.show() # 显示图像


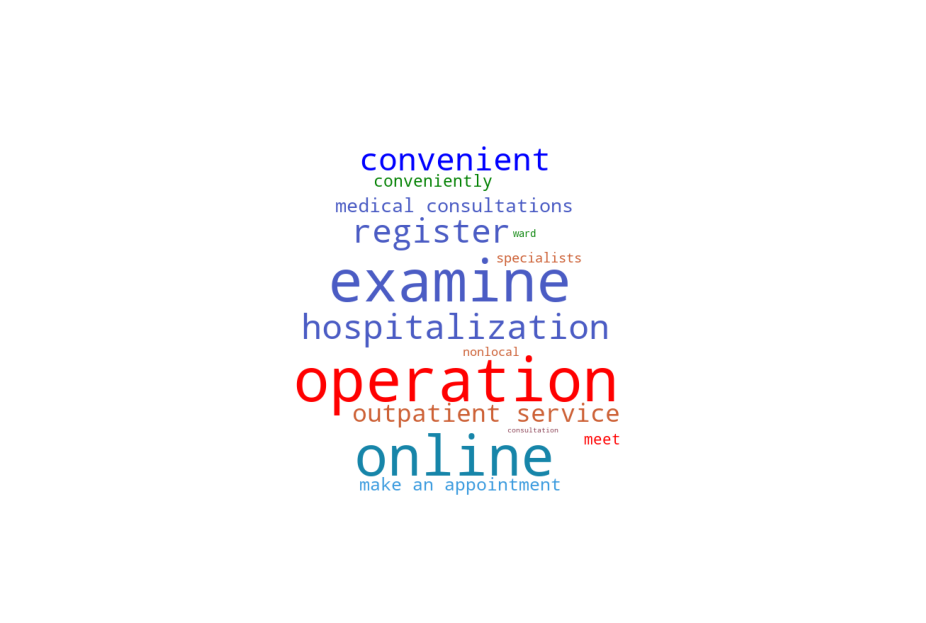


**Running result**
